# Novel Pharmacokinetic/Pharmacodynamic Parameters Quantify the Exposure–Effect Relationship of Levofloxacin against Fluoroquinolone-Resistant *Escherichia coli*

**DOI:** 10.3390/antibiotics10060615

**Published:** 2021-05-21

**Authors:** Johanna Seeger, Sebastian Guenther, Katharina Schaufler, Stefan E. Heiden, Robin Michelet, Charlotte Kloft

**Affiliations:** 1 Department of Clinical Pharmacy and Biochemistry, Institute of Pharmacy, Freie Universitaet Berlin, Kelchstr. 31, 12169 Berlin, Germany; johanna.seeger@fu-berlin.de (J.S.); robin.michelet@fu-berlin.de (R.M.); 2 Department of Pharmaceutical Biology, Institute of Pharmacy, Universitaet Greifswald, Friedrich-Ludwig-Jahn-Straße 17, 17489 Greifswald, Germany; sebastian.guenther@uni-greifswald.de; 3 Department of Pharmaceutical Microbiology, Institute of Pharmacy, Universitaet Greifswald, Friedrich-Ludwig-Jahn-Straße 17, 17489 Greifswald, Germany; katharina.schaufler@uni-greifswald.de (K.S.); stefan.heiden@uni-greifswald.de (S.E.H.)

**Keywords:** pharmacokinetic/pharmacodynamic parameters, in vitro infection model, *Escherichia coli*, levofloxacin, antibiotic resistance, minimal inhibitory concentration

## Abstract

Minimal inhibitory concentration-based pharmacokinetic/pharmacodynamic (PK/PD) indices are commonly applied to antibiotic dosing optimisation, but their informative value is limited, as they do not account for bacterial growth dynamics over time. We aimed to comprehensively characterise the exposure–effect relationship of levofloxacin against *Escherichia coli* and quantify strain-specific characteristics applying novel PK/PD parameters. In vitro infection model experiments were leveraged to explore the exposure–effect relationship of three clinical *Escherichia coli* isolates, harbouring different genomic fluoroquinolone resistance mechanisms, under constant levofloxacin concentrations or human concentration–time profiles (≤76 h). As an exposure metric, the ‘cumulative area under the levofloxacin–concentration time curve’ was determined. The antibiotic effect was assessed as the ‘cumulative area between the growth control and the bacterial-killing and -regrowth curve’. PK/PD modelling was applied to characterise the exposure–effect relationship and derive novel PK/PD parameters. A sigmoidal E_max_ model with an inhibition term best characterised the exposure–effect relationship and allowed for discrimination between two isolates sharing the same MIC value. Strain- and exposure-pattern-dependent differences were captured by the PK/PD parameters and elucidated the contribution of phenotypic adaptation to bacterial regrowth. The novel exposure and effect metrics and derived PK/PD parameters allowed for comprehensive characterisation of the isolates and could be applied to overcome the limitations of the MIC in clinical antibiotic dosing decisions, drug research and preclinical development.

## 1. Introduction

Dosing optimisation is an important strategy to tackle the global threat of antimicrobial resistance and is commonly based on pharmacokinetic/pharmacodynamic (PK/PD) indices, which relate an exposure metric of an antibiotic, e.g., C_max_ or AUC, to an effect metric, the minimal inhibitory concentration (MIC) of the pathogen [1,2,3]. Limitations of the MIC value in predicting in vivo susceptibility of pathogens are well known: 2-fold dilution series of antibiotic concentrations are conventionally used, with visual evaluation after 16–20 h of incubation under standardised conditions [4]. Hence, the MIC does not account for bacterial growth dynamics over time, evaluation by the unaided eye is subjective and uncertainty of MIC determinations, comprising biological and technical uncertainty, is thus high [5,6,7]. Moreover, MIC values of a bacterial wild-type population usually cover 3–5 two-fold dilutions [8] and hence, clinical decision-making, guided by MIC-based PK/PD indices, entails a risk of inappropriate categorisation of the bacterial strain. Consequently, choice of an inefficacious antibiotic and dosing regimen can result in treatment failure. Further, MIC-guided preclinical research and development potentially misses important characteristics of the growth and kill behaviour of a bacterial strain under antibiotic exposure, which can be elucidated using time–kill curve experiments [9]. Complementarily, whole-genome sequencing (WGS) has evolved to predict the susceptibility of a bacterial strain from its genome, but it still lacks harmonisation, quality standards and clinical implications of detected genomic resistance mechanisms [10]. Phenotypic susceptibility reflects a complex interplay between chromosomal and acquired resistance mechanisms and phenotypic adaptation, such as the so-called SOS response and persister cell formation under antibiotic exposure [11,12]. Emergence of persisting bacterial subpopulations is not represented in the unchanged MIC value of these phenotypically adapted bacteria [13]. Yet, prediction of phenotypic resistance based on genotyping has not been established [14].

To comprehensively characterise the PK/PD relationship of an antibiotic and a pathogen, the European Medicines Agency suggests time–kill curve experiments utilising in vitro models in preclinical studies [15]. Under antibiotic exposure, serial bacterial concentrations are determined to assess the antibiotic effect, that is the growth, kill and regrowth behaviour, over time [9,16,17]. Thereby, the effect of multiple antibiotic concentration–time profiles (C(t) profiles) on various bacterial strains can be investigated. Static in vitro infection models employ constant drug concentrations, while dynamic in vitro infection models mimic human target-site C(t) profiles resulting from different dosing regimens and routes of administration [17]. Various PK/PD metrics derived from in vitro infection models have been proposed [1,18], but preclinical evaluation of novel drug candidates and identification of PK/PD targets mostly rely on MIC-based PK/PD indices, for example *f*AUC/MIC [19]. Like the MIC, these PK/PD metrics do not account for the bacterialkilling and regrowth curve over time or the shape of the antibiotic C(t) profile.

Aiming to take full advantage of static and dynamic in vitro infection models, the fluoroquinolone levofloxacin (LVX) and the pathogen *Escherichia coli (E. coli)* were chosen as the model compound and organism, as fluoroquinolone resistance in extra-intestinal pathogenic *E. coli* is alarming [20]. LVX is a critically important antibiotic, indicated for severe infections, such as nosocomial pneumonia and pyelonephritis [21,22]. Both C_max_/MIC ratio and AUC/MIC ratio are discussed as PK/PD indices best predicting LVX efficacy [23,24,25,26,27,28]. The European Committee on Antimicrobial Susceptibility Testing (EUCAST) refers to a target *f*AUC/MIC ratio of 72 for clinical efficacy in the current rationale for clinical breakpoints [19]. Based on a neutropenic mouse thigh model, a *f*AUC/MIC target value of 35.6 has been determined for bacteriostasis, 67.4 for 1-log_10_ reduction and 140 for 2-log_10_ reduction of bacterial load. In the present study, we aimed to discriminate the exposure–effect relationship of LVX resistant *E. coli* strains, going beyond the typically applied PD targets and exploiting the full bacterial growth, kill and regrowth trajectory. The study focused on moderately resistant strains, aiming to quantitatively discriminate the extent of observed initial reduction and regrowth of bacterial populations under LXV exposure.

Fluoroquinolone resistance mechanisms encompass mutations in the quinolone-resistance-determining regions (QRDR) of the target enzyme encoding genes *gyrA* and *parC,* and plasmid-mediated quinolone resistance, especially plasmids encoding for Qnr proteins [29,30,31,32]. Furthermore, altered membrane function affecting drug permeation contributes to reduced fluoroquinolone susceptibility, e.g., decreased expression of porin diffusion channels in Gram-negative bacteria or increased expression of efflux transporters [29,30]. The impact of genomic fluoroquinolone resistance mechanisms on phenotypic resistance is relatively well investigated [10,31,32], but clinical implications regarding fluoroquinolone dosing are missing.

This study aimed to comprehensively characterise the exposure–effect relationship of LVX against *E. coli* based on static and dynamic in vitro infection model experiments. Bacterial growth and kill behaviour over time as a consequence of the full antibiotic C(t) trajectory was quantified by novel PK/PD metrics and discussed in the light of the genomic characteristics of clinical *E. coli* isolates.

## 2. Results

### 2.1. Characterisation of Bacterial Strains

Three clinical *E. coli* isolates were identified as sequence types (STs) 58, ST88 and ST167. Mutations in QRDR and acquired fluoroquinolone resistance genes were identical before and after exposure in static and dynamic in vitro infection model experiments (Table 1). MIC values classified the three isolates as LVX-resistant according to EUCAST [33].

### 2.2. In Vitro Infection Model Experiments

All *E. coli* isolates displayed a strain-specific initial reduction of bacterial concentrations, followed by regrowth under exposure to LVX concentrations ≤ 2-fold the MIC of the isolate in static in vitro infection model experiments [34].

In the dynamic in vitro infection model, LVX C(t) profiles resulting from a 750 mg, 90 min intravenous (i.v.) infusion in humans were successfully mimicked based on a two-compartment PK model [35]. Experimentally mimicked LVX C(t) profiles were comparable between the strains and did not show a trend towards lower or higher exposure for any of the isolates, but initial killing and regrowth within 24 h were observed with a strain-specific extent, even for two isolates sharing the same MIC value of 8 mg/L (ST58 and ST167) [34].

### 2.3. Pharmacokinetic/Pharmacodynamic Metrics

Exposure was quantified by the cumulative area under the LVX–C(t) curve (cumAUC), which increased linearly over time for static IVIM experiments (Supplementary Figure S1, bottom). Differently, for the dynamic exposure pattern, steeply increasing LVX concentrations in the beginning, and decreasing LVX concentrations in the terminal part of a C(t) profile were represented in a sigmoidal LVX cumAUC–time trajectory (Figure S1, top). Maximum LVX cumAUC(t) values, reached at the end of each experiment, were approximately 7-fold higher for static compared to dynamic exposure (1536 vs. 216 mg·h·L^−1^, respectively).

A novel effect metric was derived based on the area between the growth-control (GC) and the bacterial-killing and -regrowth curve (ABBC), introduced by Firsov et al. [36]. In our study, the ABBC was determined cumulatively over time of exposure (cumABBC(t)) and normalised to the cumulative area under the GC curve at that timepoint (cumAUGC(t)) to account for growth without antibiotic exposure as baseline and to distinguish between the growth and kill behaviour of the isolates. The maximum normalised cumABBC(t) was observed at the timepoint of the minimum bacterial concentrations, before observing regrowth.

In the static in vitro infection model, the maximum normalised effect was similar for the investigated isolates (ST58 = 0.818, ST88 = 0.852, ST167 = 0.858, Figure S2, right). Contrary, under dynamic LVX exposure, the maximum normalised effect quantitatively demonstrated differences in bacterial growth and kill behaviour of the strains: ST58 = 0.377, ST167 = 0.627 and ST88 = 0.706 (Figure S2, left). Hence, the normalised cumulative effect was only 1- to 2-fold higher in the static compared to the dynamic in vitro infection model, despite the 7-fold higher cumulative exposure.

### 2.4. Exposure–Effect Relationship

Relating the dynamic PK metric cumAUC(t) to the dynamic PD metric normalised cumABBC(t), initially sigmoidally increasing exposure–effect curves were observed for static and dynamic LVX exposure, and regrowth was represented by a decline in the normalised cumABBC(t) at higher exposure (Figure S3). For the static exposure, this decline was less pronounced (smaller slope) than for dynamic exposure, probably as a consequence of the linearly increasing cumAUC(t) for static LVX concentrations, compared to decreasing incremental LVX AUC in the terminal part of the dynamic C(t) profiles.

A sigmoidal E_max_ model with an additional inhibition term best characterised the observed exposure–effect relationship for the three *E. coli* isolates under static and dynamic LVX exposure:

$$Effect=\frac{cumABBC\left(t\right)}{cumAUGC\left(t\right)}=\frac{\;cumAUC{\left(t\right)}^{n}}{cumAUC_{50}^{n}+cumAUC{\left(t\right)}^{n}}∙\;\frac{1}{1+\frac{cumAUC\left(t\right)}{cumAUC_{reg}}}$$

In the PK/PD model, the cumABBC/cumAUGC ratio quantified the antibiotic effect as a function of time, where cumAUC(t) was the cumulative exposure at the timepoint t, the parameter cumAUC_50_ was the cumAUC causing 50% of the maximum effect, cumAUC_reg_ was the cumAUC at which regrowth occurred, and the so-called “Hill factor”(n) quantified the steepness of the exposure–effect relationship.

**Figure 1:**
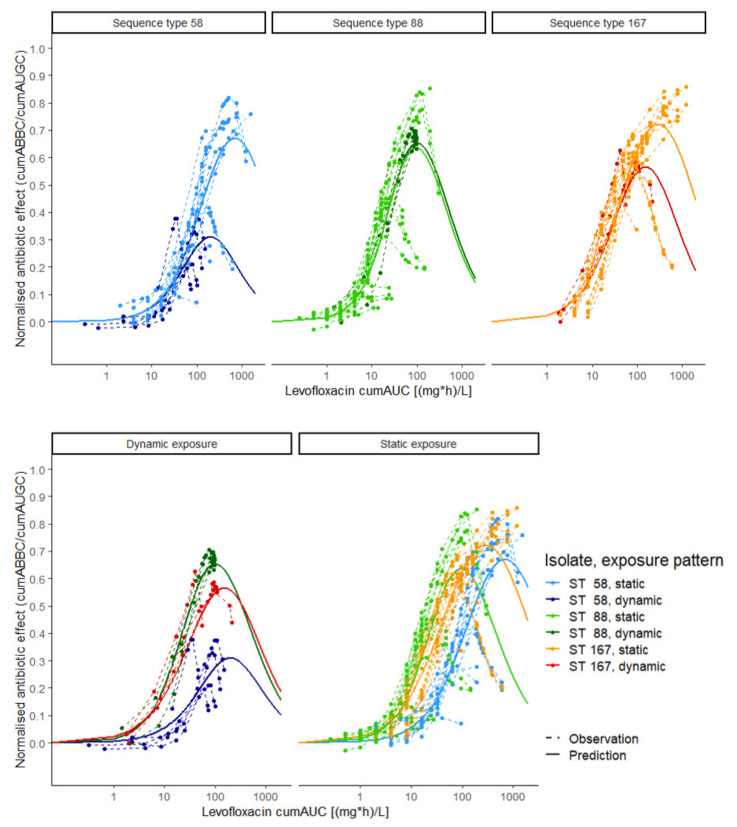
Exposure–effect relationship of levofloxacin against *Escherichia coli* in static and dynamic in vitro infection model experiments. Exposure was determined as the cumulative area under the levofloxacin concentration–time curve (cumAUC(t)); effect was determined as the cumulative area between the growth control and the bacterial killing and regrowth curve (cumABBC(t)), normalised to the area under the growth control curve (cumAUGC(t)); observations (points and dashed lines) and predictions (solid lines) were based on the E_max_ model with inhibition term. Colours: three *Escherichia coli* isolates under dynamic (dark green, red and blue) and static (light green, orange and light blue) exposure; upper panel: exposure–effect relationship per isolate; lower panel: exposure–effect relationship per exposure pattern; ST: sequence type.

Here, the sigmoidally increasing effect in the first part of the exposure–effect course (Figure 1) was primarily determined by the cumAUC(t) causing 50% of the maximum effect (cumAUC_50_), with a steeper increase in effect for a lower cumAUC_50_ estimate. Bacterial regrowth, represented by a reverse effect (i.e., decrease in antibiotic effect) at higher exposure at later time points, was determined by the LVX cumAUC(t) causing regrowth (cumAUC_reg_)—small cumAUC_reg_ estimates represented regrowth at lower cumulative LVX exposure, while large cumAUC_reg_ estimates resulted in a negligible impact of the inhibition term and therefore reduced the PK/PD model to a simple sigmoidal E_max_ model (i.e., only the left part of the equation). For each isolate, cumAUC_50_ and the Hill factor (n) were jointly estimated for static and dynamic exposure, while cumAUC_reg_ was estimated separately (cumAUC_reg,static_, cumAUC_reg,dynamic_).

Unlike static LVX concentrations, the predicted exposure–effect relationship in the dynamic experimental setting (Figure 1, dark blue, dark green and red solid lines) reflected the clinical situation, as dynamic C(t) profiles were mimicked using a PK model developed based on clinical data [35]. The reversed effect at higher exposure values indicated the inappropriateness of the approved dosing regimen, discerning between clinical isolates sharing the same MIC value (ST58 and ST167, dark blue and dark red solid lines).

#### 2.4.1. Stratification per *E.coli* Strain

The observed differences between the strains in initial bacterial reduction were quantified by their cumAUC_50_ estimates, being smallest for ST88, followed by ST167 (almost 2-fold higher), and being largest for ST58 (more than 5-fold higher compared to ST88, Table 2), indicating the highest LVX susceptibility for ST88, in line with the lower MIC value of this isolate (2 mg/L). However, differences in the exposure–effect relationship between ST58 and ST167 sharing the same MIC value (8 mg/L) were observed—the initial bacterial reduction was less pronounced for ST58, which was quantified by a more than 3-fold higher cumAUC_50_ estimate compared to ST167 (158 vs. 49.4 mg·h·L^−1^).

CumAUC_reg_ estimates revealed strain-dependent differences between the exposure patterns—the cumAUC_reg,static_ estimate was smallest for ST88, followed by ST167 (5-fold higher) and ST58 (9.5-fold higher compared to ST 88), being in line with the order of the cumAUC_50_ estimates. However, the cumAUC_reg,dynamic_ estimate was smallest for ST58, followed by ST88 and ST167. Comparing the static setting with constant LVX exposure to the dynamic setting with clinically relevant LVX C(t) profiles, the cumAUC_reg,static_/cumAUC_reg,dynamic_ ratio indicated the tendency of an isolate to show regrowth preferably in the static setting for a ratio < 1 and in the dynamic setting for a ratio > 1 (Table 2).

#### 2.4.2. Stratification for Static and Dynamic Exposure

To further elucidate the impact of the static LVX concentration on the exposure–effect relationship, parameters were estimated stratified per exposure pattern (static or dynamic exposure) and MIC-normalised LVX concentration for static exposure (Table S1). For ST58 and ST167, cumAUC_50_ estimates were comparable between dynamic exposure and static exposure to 1-fold MIC, while the cumAUC_50_ estimates for 2-fold MIC exposure were much higher for these isolates. Differently, for ST88, the cumAUC_50_ value was 1.6-fold higher for the dynamic exposure pattern compared to 2-fold MIC exposure (32.2 mg·h·L^−1^ vs. 19.7 mg·h·L^−1^). CumAUC_reg_ estimates were beyond the maximum observed exposure for ST58 and ST167 under static exposure to LVX concentrations of 2-fold MIC (Figure S1, bottom). Differently, for ST88, cumAUC_reg_ was 1.5-fold higher under dynamic exposure compared to static exposure to 2-fold MIC and more than 20-fold higher compared to static exposure to 1-fold MIC, respectively, indicating a tendency of the isolate to display regrowth under static rather than under dynamic exposure.

**Figure 2:**
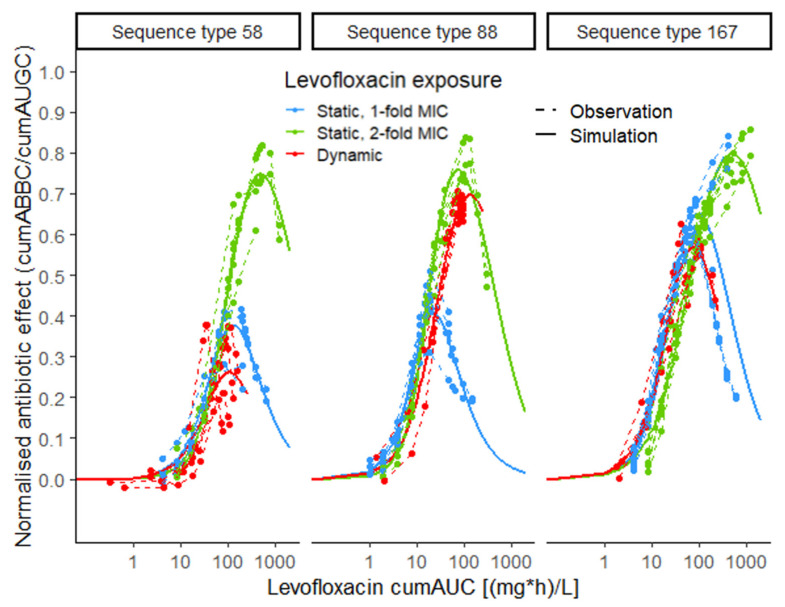
Exposure–effect relationship of levofloxacin against *Escherichia coli* in static and dynamic in vitro infection model experiments, stratified per exposure pattern (blue: static, 1-fold MIC; green: static, 2-fold MIC; red: dynamic); exposure metric: cumulative area under the levofloxacin-concentration–time profile (cumAUC(t)); effect metric: cumulative area between the growth-control and the bacterial-killing and -regrowth curve (cumABBC(t)), normalised to the area under the growth-control curve (cumAUGC(t)); observations (points and dashed lines) and deterministic simulations (solid lines) based on the E_max_ model with inhibition term; MIC: minimal inhibitory concentration.

Comparing the predicted maximum effect (E_max_), based on parameter estimates for the three isolates stratified per exposure pattern (static exposure to 1-fold MIC, 2-fold MIC and dynamic exposure), showed similar E_max_ values for ST88 under static exposure to 2-fold MIC and dynamic exposure (0.757 vs. 0.696), but requiring almost 2-fold higher LVX exposure in the dynamic setting (Table S2, Figure 2). For ST58, the maximum effect under dynamic exposure was much smaller than that from static exposure to 1-fold MIC, with only 18.6% lower cumulative LVX exposure. At the same time, LVX exposure at the maximum effect was 4.6-fold higher comparing exposure to 2-fold MIC in the static to the dynamic setting. For ST167, E_max_ was smaller in the dynamic setting compared to static exposure to 1-fold MIC (0.567 vs. 0.650), with a 22.9% smaller exposure at the maximum effect. Consequently, for ST58 and ST167, insufficient exposure in the dynamic setting might have contributed more to regrowth compared to ST88.

**Figure 3:**
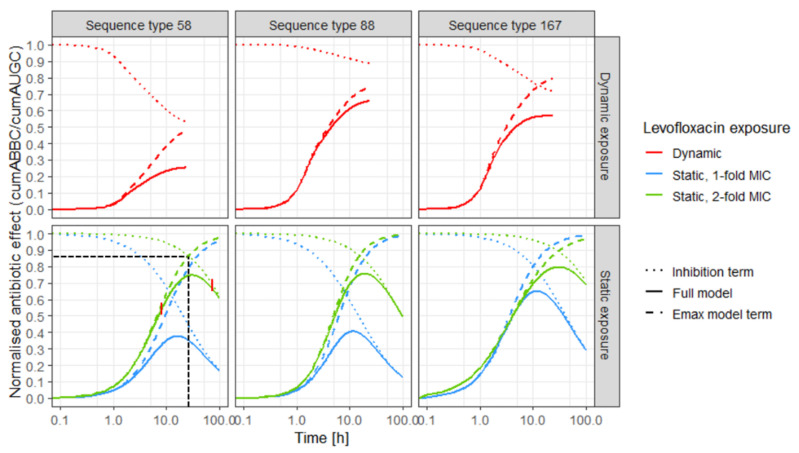
Predicted effect (solid lines) of levofloxacin over time against three *Escherichia coli* isolates (left: sequence type 58, middle: sequence type 88, right: sequence type 167) in in vitro infection model experiments, based on the E_max_ model with inhibition term; and predictions based only on the separate inhibition term (regrowth process, dashed lines) and only on the E_max_ model term (killing process, dotted lines); upper panel: predictions for dynamic exposure (red); lower panel: predictions for static exposure to 1-fold MIC (blue) and 2-fold MIC (green), black horizontal and vertical dashed lines indicate the intersection between killing and regrowth trajectories, exemplified for sequence type 58, static 2-fold exposure; vertical red lines indicate three phases of exposure–effect relationship: “killing phase”, “transition phase” and “ regrowth phase”; MIC: minimal inhibitory concentration.

The separate trajectories of the killing process, described by the E_max_ model term, and the regrowth process, characterised by the inhibition term, demonstrated the changing impact of the two processes determining the exposure–effect relationship and unveiled the relation between the two parameters, cumAUC_50_ and cumAUC_reg_ and the full normalised effect (Figure 3, Table 3).

Three phases of the effect–time trajectories were identified: In the first phase, the killing process predominantly (up to 95%) determined the effect, illustrated by the overlapping trajectories of the E_max_ model term and the full model (Figure 3, coloured dashed and solid lines). The second phase (transition phase) was determined by the two opposing processes and comprised the intersection of the killing and the regrowth trajectories (Table 3; Figure 3: exemplified for ST58 under static exposure to 2-fold MIC, black horizontal and vertical dashed lines). In the third phase, regrowth predominantly (up to 95%) determined the effect (Figure 3: overlapping dotted and solid lines). With increasing impact of the inhibition term, the effect was reduced to a strain-specific extent. Differences between the strains were more pronounced in the dynamic setting than under static exposure. The predicted E_max_ was influenced by both the steepness of the effect–time trajectory and the slope of the inhibition term. E_max_ was similar for the isolates under static exposure to LVX concentrations of 2-fold MIC, but different in the dynamic setting. The predicted effect increased later under exposure to 1-fold MIC (blue solid lines) compared to 2-fold MIC (green solid lines). Additionally, effect–time curves were steeper for exposure to static LVX concentrations of 2-fold MIC compared to 1-fold MIC. The impact of the inhibition term, reversing the effect at later time points, was more pronounced for static exposure to 1-fold MIC compared to 2-fold MIC. Under exposure to dynamic LVX concentrations, the impact of the inhibition term was most pronounced for ST58 and smallest for ST88.

## 3. Discussion

The novel time-dependent exposure and effect metrics cumAUC(t) and cumAUGC(t)-normalised cumABBC(t), respectively, were successfully applied to characterise the exposure–effect relationship of LVX against *E. coli* in a mechanism-based PK/PD model, based on experiments in two different in vitro infection models. This large experimental database, comprising static and dynamic experiments, allowed for distinction between the (re)growth and kill processes and precise estimation of easily interpretable PK/PD parameters. The derived PK/PD parameters, cumAUC_50_ and cumAUC_reg_, allowed for comparison and discrimination between the clinical isolates, even when sharing the same MIC value, as well as between static and dynamic drug exposure. Isolate-specific parameter estimates elucidated the contribution of genomic resistance and phenotypic adaptation to bacterial regrowth.

E_max_ models have been widely used to describe the relationship between an antibiotic concentration and the resulting effect [37,38,39,40,41]. Here, by implementation of cumAUC(t), the concept was extended by leveraging a metric representing the full exposure–time trajectory instead of a single C(t). Based on the approach introduced by Firsov et al., we went beyond the MIC and introduced novel parameters, capturing the different processes constituting the antibiotic effect, i.e., killing and regrowth under antibiotic exposure over time—initially, the sigmoidal E_max_ model term dominated the exposure–effect relationship, while the impact of the inhibition term increased at higher cumulative exposure values at later timepoints. CumAUC_50_ represented the sigmoidally increasing effect at low exposure, i.e., the strain-specific extent of initial bacterial reduction, while cumAUC_reg_ reflected the tendency of an isolate to regrow, with lower cumAUC_reg_ estimates for regrowth at lower exposure.

Mainly dominating the exposure–effect relationship for initially small cumAUC(t) values, the impact of previous exposure on cumAUC_50_ was small compared to the impact of previous exposure on the regrowth parameter cumAUC_reg_. Hence, for the static and the dynamic exposure pattern, a joint cumAUC_50_ value was rather determined by “inherent” characteristics of an isolate being present prior to antibiotic exposure, i.e., genomic resistance mechanisms. For optimised antibiotic dosing, prevention of bacterial adaptation causing regrowth is equally important. For that purpose, the regrowth parameter cumAUC_reg_, providing insights in the cumulative exposure causing regrowth, should be leveraged. In future, applying cumAUC_reg_ for various bacterial strains can facilitate the definition of a cumAUC_reg_ value preventing regrowth in vitro as a MIC-independent PK/PD target. With increasing impact for higher cumulative exposure, cumAUC_reg_ represented bacterial regrowth mechanisms, being different between static and dynamic exposure. Stratified predictions for the investigated exposure patterns (static, 1-fold MIC, 2-fold MIC and dynamic exposure) demonstrated the larger impact of the inhibition term under static compared to dynamic exposure. The intersection of the killing and regrowth trajectories indicated the time point when regrowth started to dominate the exposure–effect relationship, which was only reached for ST167 under the dynamic experimental conditions. Hence, decreasing LVX concentrations might contribute to regrowth in the dynamic exposure setting, while bacterial adaptation mechanisms, such as persister cell formation, might play a role in the strain- and exposure-pattern-dependent differences of cumAUC_reg_ estimates for static exposure. For both experimental settings, no novel genomic resistance mechanisms were detected after LVX exposure (Table 1), indicating that phenotypic adaptation mechanisms, such as persister formation, might have caused regrowth.

The cumAUC_reg,static_/cumAUC_reg,dynamic_ ratio quantitatively demonstrated the tendency to preferentially show regrowth under exposure to static LVX concentrations for ST88. As decreasing antibiotic concentrations were not present under static exposure and could thus not affect bacterial regrowth, persister cell formation might have contributed more to regrowth for ST88 compared to the other isolates. This finding indicated the superiority of continuously high LVX concentrations in the static in vitro infection model for ST58 and ST167, which can be achieved by prolonged infusion durations in the clinics. Further, the developed PK/PD model can be applied in a clinical setting by linking cumAUC values of patient-derived C(t) profiles to clinical outcome parameters to derive PK/PD target values.

Moreover, cumAUC_50_ and cumAUC_reg_ values enabled a more comprehensive characterisation of the exposure–effect relationship compared to MIC-based strategies. A paradigm shift towards MIC-independent PK/PD targets is highly needed, and hence, the novel PK/PD parameters present a promising framework for rational antibiotic dosing strategies, but also for ranking new antibiotics in preclinical research and development, enabling full exploitation of static and dynamic time–kill curve data.

Several studies investigated the impact of *gyrA* and *parC* mutations and *qnr* plasmids on phenotypic fluoroquinolone resistance, linking the level of phenotypic resistance to genomic properties of the investigated isolates solely with respect to MIC values [29,30,31,32]. In general, the MIC values were higher for a higher number of QRDR mutations. LVX resistance at MIC ≥ 8 mg/L has been observed in *E. coli*, if more than one *gyrA* mutation or an additional *parC* mutation was detected, while a single *gyrA* mutation normally causes LVX resistance at a low level (0.25 mg/L ≤ MIC < 8 mg/L) [32]. Plasmid-mediated quinolone resistance alone usually is not capable of elevating LVX MIC above the clinical resistance breakpoint of MIC > 1 mg/L [30,31,32].

In the current study, LVX resistance quantified by the novel PK/PD parameters cumAUC_50_ and cumAUC_reg_ indicated the highest susceptibility for ST88, followed by ST167 and the highest level of resistance for ST58. The higher number of mutations of ST167 partly explained this finding, indicating lower susceptibility compared to ST88 with one *gyrA* mutation and *qnrS* plasmids (Table 1). The elevated resistance level of ST58 was unexpected with regard to the single *gyrA* mutation of the isolate, indicating that phenotypic adaptation mechanisms, such as persister cell formation, might have contributed. Furthermore, fitness costs due to the higher number of mutations harboured by ST167 compared to ST58 and ST88 might have contributed to delayed regrowth of the isolate [42].

As for all in vitro approaches, the direct transferability of the presented results to the in vivo situation is limited and serves as a first basis, as not all relevant processes are represented in the experimental setup. Importantly, the contribution of the human immune system to bacterial killing was not captured in the applied in vitro infection models [43]. Furthermore, the method used for bacterial quantification did not detect non-cultivable persister cells. Thus, experiments to further elucidate the contribution of bacterial adaptation mechanisms to regrowth are highly warranted. Yet, in vitro approaches allow for systematic investigations under standardised conditions. The benefit of the suggested PK/PD metrics and derived PK/PD parameters was presented for LVX as a model compound and a limited number of three clinical *E. coli* isolates. To assess their external validity, these metrics should be applied to a larger number of bacterial species and antibiotics. In particular, applicability of the parameters for antibiotics with a time-dependent PK/PD relationship, such as beta-lactam antibiotics, should be explored.

## 4. Materials and Methods

### 4.1. Characterisation of Bacterial Strains

#### 4.1.1. Genotypic Resistance

Three fluoroquinolone-resistant clinical *E. coli* isolates, obtained from Charité University Medicine Berlin, were investigated. Genomic resistance mechanisms of the isolates were determined prior to LVX exposure and after representative experiments (*n* = 1 per isolate and experimental setting). Single bacterial colonies were scratched from freshly prepared overnight cultures on Columbia agar (Carl Roth GmbH, Karlsruhe, Germany) to ensure a genetically homogenous *E. coli* population. After overnight incubation in CAMHB (Oxoid GmbH, Wesel, Germany), bacterial DNA was extracted using a bacterial DNA extraction kit (GF-1, GeneOn^®^, Ludwigshafen, Germany). WGS was performed using the Illumina^®^ technology. The STs were determined and relevant QRDR mutations and acquired resistance genes were identified by applying multilocus sequence typing (MLST), utilising ResFinder 3.2, and the MLST-2.0 online tool, (Center for Genomic Epidemiology, Lyngby, Denmark), respectively [44,45].

#### 4.1.2. Antimicrobial Susceptibility Testing

For MIC determination of LVX against the investigated isolates, the broth microdilution method according to CLSI was applied [4]. Using CAMHB as growth medium, the assay was carried out twice for each isolate (*n* = 6 replicates per experiment and LVX concentration).

### 4.2. In Vitro Infection Model Experiments

To investigate bacterial growth and kill behaviour of *E. coli* over time under LVX exposure, static and dynamic time–kill curve experiments were performed as described previously [34]. In short, in the static in vitro infection model, exponential *E. coli* cultures were exposed to static LVX concentrations between 0.25- and 8-fold their MIC value for 1–3 days. In the dynamic in vitro infection model, LVX C(t) profiles resulting from a 750 mg, 90 min i.v. infusion in humans were mimicked using a continuous dilution model, which was developed based on Löwdin et al. [46]. Overall, 43 static and 12 dynamic replicates of IVIM experiments were included in the analysis.

### 4.3. Pharmacokinetic/Pharmacodynamic Metrics

CumAUC was chosen as exposure metric accounting for both time of exposure and the shape of the C(t) profile, aiming to characterise the exposure–effect relationship of LVX against the *E. coli* isolates. CumAUC was determined as a function of time (cumAUC(t)), with time starting from 0 (LVX administration) to the end of the experiment. For the static in vitro infection model, cumAUC(t) was calculated based on nominal LVX concentrations.

**Figure 4:**
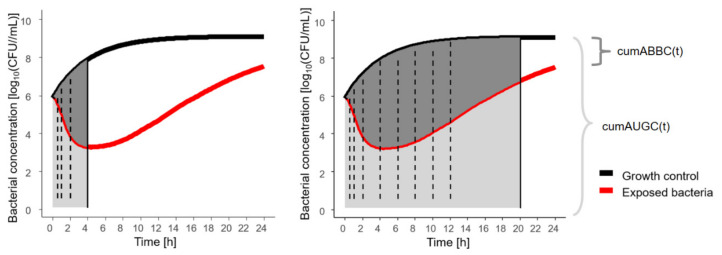
Illustration of novel pharmacodynamic metric, cumABBC(t), to quantify the antibiotic effect based on in vitro infection model experiments, exemplified for two sampling time points at 4 h (**left**) with cumABBC(4 h), and at 20 h (**right**) with cumABBC(20 h); solid vertical line: sampling time point of assessment; dashed vertical lines: intermediate sampling time points; cumABBC(t): cumulative area between growth-control and bacterial-killing and -regrowth curve as function of time (dark grey area); cumAUGC(t): cumulative area under the growth-control curve as function of time (sum of light + dark grey area).

CumABBC was determined by calculating the ABBC cumulatively over time, as cumABBC(t), realised by computing the difference between the cumABBC and the cumAUGC. To account for the changing growth dynamics of unexposed bacteria, cumABBC(t) was normalised to cumAUGC(t), representing disease progression without antibiotic treatment (Figure 4). Thereby, the effect metric was transformed to a scale between 0 (natural growth without antibiotic effect) and 1 (bacterial eradication). All cumulative areas were determined by trapezoidal integration with linear interpolation using the ‘cumtrapz’ function (R package ‘pracma’, R Foundation for Statistical Computing, Vienna, Austria).

### 4.4. Exposure–effect Relationship

#### 4.4.1. Stratification per *E. coli* Strain

The novel dynamic PK/PD metrics were applied to graphically explore the exposure–effect relationship of LVX against *E. coli* in the static and dynamic in vitro infection model. Based on these, a PK/PD model was developed to derive parameters characterising the exposure–effect relationship. Different mathematical implementations, e.g., ordinary and sigmoidal E_max_ models combined with different inhibition terms, linking cumAUC(t) to cumAUGC(t)–normalised cumABBC(t), were investigated. Nonlinear regression (‘optim’ function, R package ‘deSolve’ R Foundation for Statistical Computing, Vienna, Austria) was performed applying the Nelder–Mead and the conjugate gradient algorithm in two consecutive steps of the minimisation process. Models were compared based on precision of parameter estimates, extent of proportional residual variability and Akaike information criterion [47]. Isolate-specific PK/PD parameters were estimated.

#### 4.4.2. Stratification for Static and Dynamic Exposure

To explore the nature of the exposure–effect relationship further, parameter estimation was performed stratifying per MIC-normalised LVX exposure (for static, 1-fold MIC and 2-fold MIC, and dynamic exposure pattern). Deterministic simulations were performed for each strain and exposure pattern, the maximum predicted effect (E_max_) and the corresponding cumulative LVX exposure (cumAUC(t)) were determined. The property of an isolate to regrow preferentially under exposure to dynamic C_LEV_ was quantified by the ratio between the cumAUC value causing regrowth under static LVX exposure (cumAUC_reg,static_) and the cumAUC value causing regrowth under dynamic LVX exposure (cumAUC_reg,dynamic_) for each strain. The contribution of the two model parameters, cumAUC_50_ and cumAUC_reg_, to the effect–time trajectories was assessed graphically by plotting the E_max_ model and the inhibition term separately as a function of time for the three isolates and exposure patterns (static, 1-fold MIC and 2-fold MIC, and dynamic exposure). The time and exposure of increasing impact of the inhibition term was determined as ≥5% deviation between the trajectory of the E_max_ model term and the full model (Table 3, Figure 3, red vertical lines). The time point of full dominance of the inhibition term was defined as ≤5% deviation between the full model and the inhibition term.

## 5. Conclusions

In this study, a large experimental database, comprising static and dynamic in vitro infection model experiments, was analysed with in silico PK/PD modelling, leading to novel antibiotic exposure and effect metrics. Differences between static and dynamic exposure regarding the exposure–effect relationship, quantified by derived PK/PD parameters, highlighted the limitations of the static in vitro infection model to appropriately reflect the in vivo setting. In dynamic in vitro infection model experiments, regrowth of the investigated clinical isolates was observed under exposure to clinically relevant LVX C(t) profiles, indicating the inappropriateness of the approved dosing regimen. The applied PK/PD parameters allowed for discrimination between two isolates sharing the same MIC, but different genomic characteristics. Comparing the exposure–effect relationship of different exposure patterns demonstrated the relevance of the shape of the antibiotic C(t) profile. The introduced PK/PD metrics and PK/PD parameters present a promising framework for ranking new antibiotics in drug research and development, to more comprehensively characterise PK/PD relationships, investigate the adequateness of proposed or established dosing regimens and overcome the limitations of the MIC.

## Figures and Tables

**Table 1:** Sequence types, mutations in quinolone-resistance-determining regions, acquired fluoroquinolone resistance mechanisms and levofloxacin minimal inhibitory concentrations of three investigated *Escherichia coli* isolates, before and after levofloxacin exposure, in in vitro infection model experiments.

Sequence Type	Mutations in QRDR		Acquired Fluoroquinolone Resistance	Minimal Inhibitory Concentration (mg/L)
	*gyrA*	*parC*		
58	Ser-83→Leu	WT	-	8
88	Ser-83→Leu	WT	*qnrS1*	2
167	Ser-83→Leu Asp-87→Asn	Ser-80→Ile	-	8

QRDR: quinolone-resistance-determining regions; *gyrA* and *parC:* genes encoding for subunits of bacterial gyrase and topoisomerase IV; Ser: serine; Leu: leucine; Asp: aspartic acid; Ile: isoleucine; Asn: asparagine; WT wild type; *qnrS1*: quinolone resistance plasmid S1; “→” indicates replacement of amino acid.

**Table 2:** Parameter estimates and parameter imprecision of a sigmoidal E_max_ model combined with an inhibition term, describing the exposure–effect relationship of levofloxacin against three clinical *Escherichia coli* isolates in static and dynamic in vitro infection model experiments.

Parameter (unit)	Estimate (RSE, %)		
	Sequence Type 58	Sequence Type 88	Sequence Type 167
cumAUC_50_ (mg·h·L^−1^)	158 (9.45)	28.6 (7.85)	49.4 (7.54)
Hill	1.02 (5.49)	1.25 (5.37)	0.961 (6.62)
cumAUC_reg, static_ (mg·h·L^−1^)	3132 (36.5)	330 (22.9)	1679 (20.3)
cumAUC_reg, dynamic_ (mg·h·L^−1^)	248 (34.6)	373 (34.9)	473 (39.2)
cumAUC_reg,static_/cumAUC_reg,dynamic_ ratio	12.6	0.885	3.55
Proportional residual variability, % CV	4.00 (11.8)	4.33 (11.7)	3.33 (11.3)

RSE: relative standard error (imprecision of parameter estimates); cumAUC_50_: exposure, determined as cumulative area under the levofloxacin concentration–time curve, causing 50% of the maximum effect; cumAUC_reg, static_: exposure causing regrowth in a static in vitro infection model; cumAUC_reg_,_dynamic_: exposure causing regrowth in a dynamic in vitro infection model; Hill: Hill factor (steepness of exposure–effect relationship); CV: coefficient of variation.

**Table 3:** Dominance * of killing and regrowth processes in the three different phases of the effect–time trajectories (see Figure 3, red vertical lines in lower left panel) of three *Escherichia coli* isolates under exposure to static levofloxacin (LVX) concentrations (1- and 2-fold the minimal inhibitory concentration (MIC) of the isolate), based on parameter estimates stratified per exposure pattern.

	Sequence Type 58		Sequence Type 88		Sequence Type 167	
C_LVX_ = 1-fold MIC	Time (h)	Effect	Time (h)	Effect	Time (h)	Effect
Dominance of killing process (Higher impact of E_max_ term)	≤1.10	≤0.0539	≤0.80	≤0.358	≤2.20	≤0.320
Intersection of killing and regrowth trajectories	13.3	0.610	8.7	0.630	10.1	0.803
Dominance of regrowth process (Higher impact of inhibition term)	≥90.0	≥0.179	≥41.0	≥0.252	≥31.4	≥0.541
**C_LVX_ = 2-fold MIC**						
Dominance of killing process (Higher impact of E_max_ term)	≤8.70	≤0.560	≤5.30	≤0.503	≤13.1	≤0.737
Intersection of killing and regrowth trajectories	26.2	0.863	15.3	0.866	29.9	0.892
Dominance of regrowth process (Higher impact of inhibition term)	≥60.0	≥0.697	≥29.5	≥0.732	≥66.0	≥0.750

* Dominance defined as ≤5% deviation from predicted trajectory based on full model (see Figure 3, red vertical lines in lower left panel).

## Data Availability

Data will be provided upon reasonable request.

## Supplementary Materials

The following are available online at https://www.mdpi.com/article/10.3390/antibiotics10060615/s1, Figure S1: Levofloxacin exposure metric, Figure S2: Antibiotic effect of levofloxacin against *Escherichia coli,* Figure S3: Exposure-effect relationship of levofloxacin against *Escherichia* coli in static and dynamic in vitro infection model experiments, Table S1: Parameter estimates and parameter imprecision of a sigmoidal E_max_ model combined with an inhibition term, Table S2: Antibiotic exposure at predicted maximum effect.
