# Supplementary material for: Novel Pharmacokinetic/Pharmacodynamic Parameters Quantify the Exposure–Effect Relationship of Levofloxacin against Fluoroquinolone-Resistant Escherichia coli
Source: Antibiotics (Basel). 2021 May 21;10(6):615. doi: 10.3390/antibiotics10060615 (PMC8224043; doi:10.3390/antibiotics10060615)
Supplement: Supplementary file 1 [file antibiotics-10-00615-s001.zip › antibiotics-1210808-supplementary.pdf]

Table S1. Parameter estimates and parameter imprecision of a sigmoidal E_{max} model combined with an inhibition term, describing the exposure-effect relationship of levofloxacin against 3 clinical *Escherichia coli* isolates in static and in the dynamic *in vitro* infection model experiments, stratified per exposure pattern (static exposure to 1-fold MIC, 2-fold MIC and dynamic exposure).

Parameter [unit]	Estimate [RSE, %]		
	C_{LVX}=1 MIC	C_{LVX}=2 MIC	C_{LVX}=dynamic
**Sequence type 58**			
$cumAUC_{50}$ [mg·h·L^{-1}]	75.6 [10.2]	106 [4.87]	83.0 [30.5]
Hill	1.31 [7.02]	1.34 [4.35]	1.28 [12.5]
$cumAUC_{reg}$ [mg·h·L^{-1}]	167 [10.8]	2643 [17.1]	87.5 [44.0]
Proportional residual variability, %CV	0.925 [22.4]	0.802 [19.8]	4.85 [24.0]
**Sequence type 88**			
$cumAUC_{50}$ [mg·h·L^{-1}]	12.6 [5.67]	19.7 [3.64]	32.2 [10.1]
Hill	1.57 [4.77]	1.65 [3.95]	1.20 [8.04]
$cumAUC_{reg,}$ [mg·h·L^{-1}]	29.7 [7.40]	397 [10.2]	615 [44.7]
Proportional residual variability, %CV	0.737 [19.8]	0.580 [20.4]	0.695 [24.0]
**Sequence type 167**			
$cumAUC_{50}$ [mg·h·L^{-1}]	28.9 [8.71]	64.6 [4.66]	27.2 [8.45]
Hill	1.36 [8.92]	1.05 [4.62]	1.28 [7.64]
$cumAUC_{reg,}$ [mg·h·L^{-1}]	333 [13.5]	3956 [20.3]	195 [14.2]
Proportional residual variability, %CV	3.09 [17.9]	0.781 [17.1]	0.703 [26.2]

RSE: Relative standard error (imprecision of parameter estimates); $cumAUC_{50}$: exposure, determined as cumulative area under the levofloxacin concentration-time curve, causing 50% of the maximum effect, $cumAUC_{reg}$: exposure causing re-growth, MIC: minimal inhibitory concentration, Hill: Hill factor (steepness of exposure-effect relationship), CV: coefficient of variation.

Table S2. Antibiotic exposure at predicted maximum effect (E_{max}), determined as cumulative area-under-the-levofloxacin-concentration-time curve ($cumAUC_{max}$), and E_{max}, determined as cumulative area-between-the-growth-control-and-the-bacterial-killing-and-regrowth curve (cumABBC(t)), normalised to the area-under-the-growth-control curve (cumAUGC(t)), and cumulative exposure in 24 h (cumAUC(24 h)) for 3 *Escherichia coli* isolates, predictions based on the developed E_{max} model with inhibition term, stratified per exposure pattern: static exposure to 1-fold minimal inhibitory concentration (MIC), static exposure to 2-fold MIC and dynamic exposure to a levofloxacin concentration-time profile resulting from mimicking a 750 mg, 90 min intravenous infusion in plasma in dynamic *in vitro* infection model experiments.

	C_{LVX}=1 MIC	C_{LVX}=2 MIC	C_{LVX}=dynamic
**Sequence type 58**			
$cumAUC_{max}$ [mg·h·L^{-1}]	129	486	105
E_{max}	0.377	0.747	0.261
cumAUC(24 h) [mg·h·L^{-1}]	192	384	77.6
**Sequence type 88**			
$cumAUC_{max}$ [mg·h·L^{-1}]	23.0	76.0	136
E_{max}	0.406	0.757	0.696
cumAUC(24 h) [mg·h·L^{-1}]	48	96	77.6
**Sequence type 167**			
$cumAUC_{max}$ [mg·h·L^{-1}]	96.0	493	74.0
E_{max}	0.650	0.796	0.567
cumAUC(24 h) [mg·h·L^{-1}]	192	384	77.6

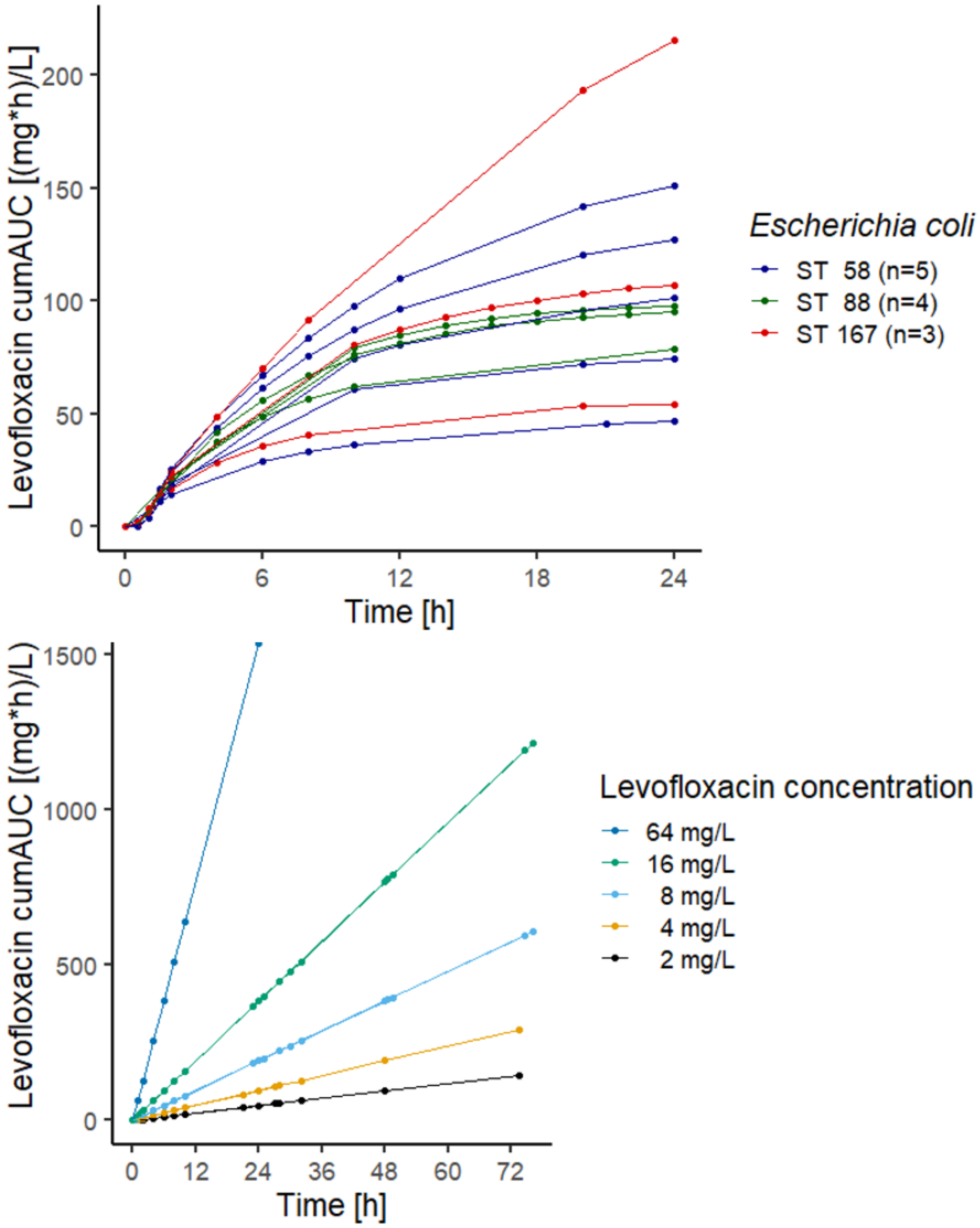

Figure S1. Levofloxacin exposure metric, determined as cumulative area-under-the-concentration-time curve (cumAUC) over time in *in vitro* infection model experiments, top: exposure metric over time, resulting from mimicking a 750 mg, 90 min intravenous infusion in plasma in **dynamic** *in vitro* infection model experiments (n=12 replicates), colours: 3 exposed *Escherichia coli* isolates, bottom: exposure metric over time in **static** *in vitro* infection model experiments, colours: nominal levofloxacin concentrations, points: sampling times, ST: sequence type.

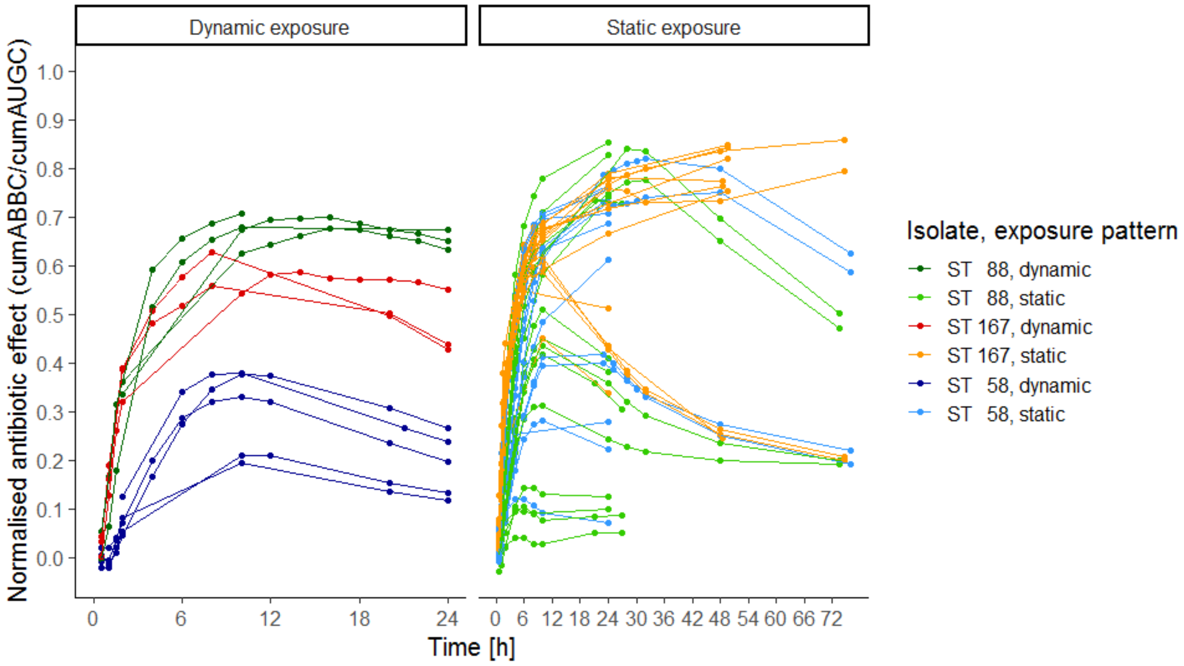

Figure S2. Antibiotic effect of levofloxacin against *Escherichia coli*, determined as the cumulative area-between-the-growth-control-and-the-bacterial-killing-and-regrowth curve (cumABBC(t)), normalised to the area-under-the-growth-control curve (AUGC(t)) of unexposed bacteria over time in *in vitro* infection model experiments; left: effect of levofloxacin concentration-time profiles resulting from mimicking a 750 mg, 90 min intravenous infusion in plasma in **dynamic** *in vitro* infection model experiments (n=12 replicates), right: effect of constant levofloxacin concentrations in **static** *in vitro* infection model experiments (n=43 replicates), colours: 3 *Escherichia coli* isolates under dynamic (dark green, red and blue) and static (light green, orange and light blue) exposure, points: sampling times, ST: sequence type.

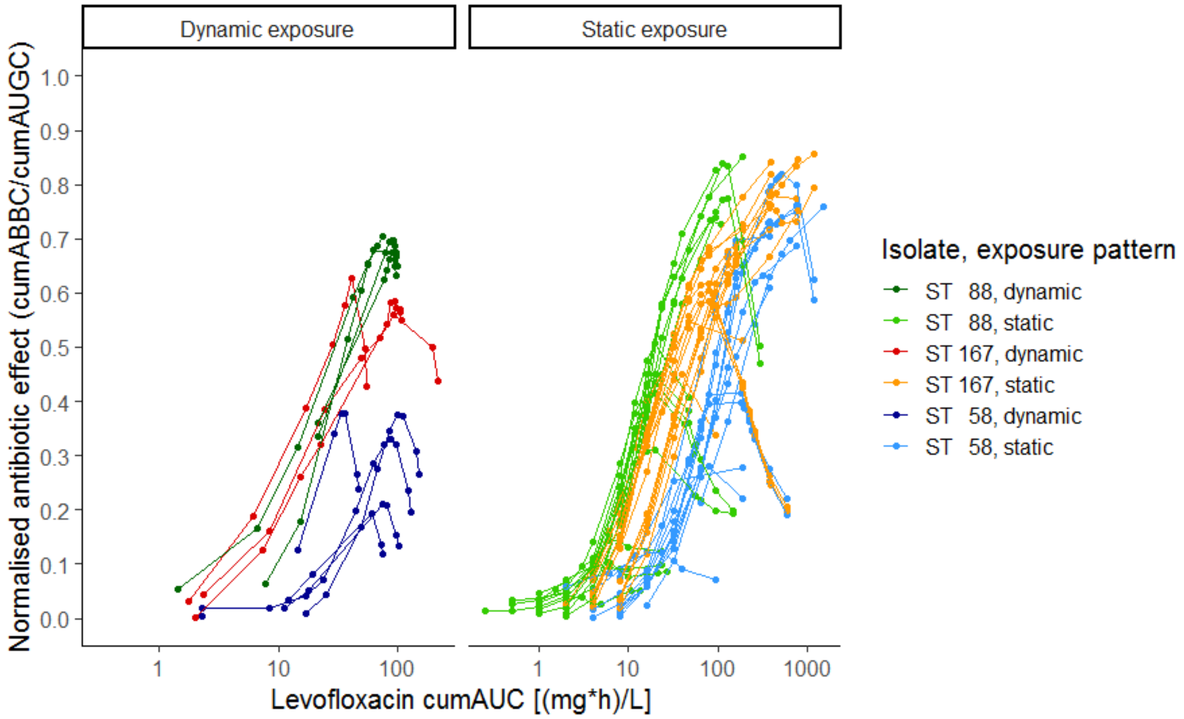

Figure S3. Exposure-effect relationship of levofloxacin against *Escherichia coli* in static and dynamic *in vitro* infection model experiments, exposure determined as cumulative area-under-the-concentration-time curve (cumAUC(t)), effect determined as cumulative area-between-the-growth-control-and-the-bacterial-killing-and-regrowth curve (cumABBC(t)), normalised to the area-under-the-growth-control curve (cumAUGC(t)), left: exposure-effect relationship resulting from mimicking a 750 mg, 90 min intravenous infusion in plasma in **dynamic** *in vitro* infection model experiments (n=12 replicates), right: exposure-effect relationship of constant levofloxacin concentrations in **static** *in vitro* infection model experiments (n=43 replicates), colours: 3 *Escherichia coli* isolates under dynamic (dark green, red and blue) and static (light green, orange and light blue) exposure, points: sampling times, ST: sequence type.
